# Isolation and characterization of a high-efficiency algicidal bacterium *Pseudoalteromonas* sp. LD-B6 against the harmful dinoflagellate *Noctiluca scintillans*

**DOI:** 10.3389/fmicb.2022.1091561

**Published:** 2022-12-22

**Authors:** Junyue Wang, Xueyao Yin, Mingyang Xu, Yifan Chen, Nanjing Ji, Haifeng Gu, Yuefeng Cai, Xin Shen

**Affiliations:** ^1^Jiangsu Key Laboratory of Marine Bioresources and Environment, Jiangsu Key Laboratory of Marine Biotechnology, Jiangsu Ocean University, Lianyungang, China; ^2^Co-Innovation Center of Jiangsu Marine Bio-Industry Technology, Jiangsu Ocean University, Lianyungang, China; ^3^Third Institute of Oceanography, Ministry of Natural Resources, Xiamen, China

**Keywords:** *Noctiluca scintillans*, harmful algal bloom, algicidal bacteria, algicidal activity, *Pseudoalteromonas*

## Abstract

The dinoflagellate *Noctiluca scintillans* is a harmful algal species that is globally distributed and poses a certain threat to marine ecosystems. Recent research has shown that the application of algicidal bacteria is a promising method to prevent and control such harmful algal blooms (HABs), given its advantages of safety and efficiency. In this study, a strain of algicidal bacterium LD-B6 with high efficiency against *N. scintillans* was isolated from the coastal waters of Lianyungang, China. 16S rDNA sequence analysis showed that the strain LD-B6 belongs to the genus *Pseudoalteromonas*. Furthermore, the algicidal effect of LD-B6 on *N. scintillans* was investigated. The results showed that strain LD-B6 exerted strong algicidal activity against *N. scintillans*. After 12 h of bacterial culture addition to algal cultures at a 2% final volume rate, the algicidal activity reached 90.5%, and the algicidal activity of LD-B6 was influenced by the density of *N. scintillans*. In addition, the algicidal bacterium LD-B6 was found to indirectly lyse algal cells by secreting extracellular compounds. These algicidal compounds were stable, indicating that they are not proteins. Importantly, strain LD-B6 was broadly general, showing varying degrees of lysing effects against five of the six algal species tested. On the basis of the described studies above, the algicidal powder was also initially developed. In summary, the isolated bacterial strain LD-B6 shows the potent algicidal capability to serve as a candidate algicidal bacterium against *N. scintillans* blooms.

## Introduction

Blooms usually refer to the phenomenon documented when some microalgae, protozoa, or bacteria over-proliferate or accumulate in aquatic environments, causing water discoloration. The international scientific community collectively refers to harmful algal blooms (HABs) as for those harmful or toxic blooms (Anderson et al., 2002; Grattan et al., 2016). In recent years, there has been a significant increase in the scale and frequency of HABs with the intensification of water eutrophication, global warming, ocean acidification, and other phenomena (Griffith and Gobler, 2020; Karlson et al., 2021; Sha et al., 2021).

The dinoflagellate *Noctiluca scintillans* is a harmful algal species with a global distribution that has caused severe HABs in many coastal waters around the world, posing a significant threat to fishery resources and marine ecosystems (Qi et al., 2019; Piontkovski et al., 2021). According to the differences in nutritional requirements, there are two forms of *N. scintillans*: Red and green (Harrison et al., 2011). Red *N. scintillans* is a heterotrophic plankton. In contrast, green *N. scintillans* is a mixoplankton that contains a photosynthetic symbiont, *Pedinomonas noctilucae* (a prasinophyte). Although *N. scintillans* does not produce phytotoxins or other toxins, its bloom secretes a large amount of mucus that adheres to fish gills and causes suffocation (Tan et al., 2002). Most importantly, it may cause hypoxia in the surrounding environment and release a high concentration of ammonia when the bloom declines, which not only impact aquaculture but also have a detrimental effect on aquatic environments (Montani et al., 1998; Miyaguchi et al., 2008; Zhang et al., 2017). In addition, the food vacuoles of *N. scintillans* have been found to contain toxigenic microalgae, suggesting that *N. scintillans* may act as a vector of phycotoxins to higher trophic levels (Escalera et al., 2007). Due to such harmful attributes of *N. scintillans* blooms, it is particularly important and urgent to carry out related research on its prevention and control.

In this regard, physical, chemical, and biological methods have been proposed for controlling the HABs (Anderson, 2009). The use of modified clay is one of the most common physical methods (Liu et al., 2016; Yu et al., 2017). The chemical approach refers to the use of foreign additives such as chemical reagents (Geciova et al., 2002; Maršálek et al., 2020). However, the implementation of chemical and physical methods for the management of HABs is still limited due to the disadvantages of high cost, poor specificity, and secondary pollution (Yu et al., 2017). Biological methods such as the use of algicidal bacteria have been widely studied in the control of HABs because of their advantages of safety and efficiency (Wang et al., 2020; Coyne et al., 2022). Studies have shown that about 50% of the algicidal bacteria belong to the Cytophaga/Flavobacterium/Bacteroidetes (CFB) group, and about 45% belong to Gammaproteobacteria. The remaining bacteria are from the Gram-positive genera *Micrococcus*, *Bacillus*, and *Planomicrobium* (Roth et al., 2008). Algicidal bacteria generally attack the target algae through two strategies: directly by being in contact with algal cells and indirectly by competition or secretion of extracellular active compounds by the bacteria (Azam, 1998). The mechanisms associated with algicidal bacteria mainly include a few pathways, like the destruction of cell structure, the change in enzyme activity, and the influence of algal photosynthesis or respiration (Zhang et al., 2020a; Chen et al., 2022). A large number of highly efficient algicidal bacteria have been recently isolated from various eutrophic regions (Lu et al., 2016; Zheng et al., 2018; Li et al., 2022). However, few studies have isolated algicidal bacteria against *N. scintillans* despite the potential impacts discussed above. At present, only *Marinobacteria salsuginis* BS2 (Gammaproteobacteria) has been documented to show algicidal activity against *N. scintillans* (Keawtawee et al., 2011).

In this study, the isolation of an efficient *N. scintillans* algicidal bacterium from the coastal waters of Lianyungang, China, is reported. The strain was identified by 16S rDNA sequence analysis. Its algicidal effects against red *N. scintillans* were also explored. We have also developed a preliminary algicidal powder based on the nascent research for future *N. scintillans* algal bloom management.

## Materials and methods

### *Noctiluca scintillans* cultures

The red *N. scintillans* strain was originally isolated from the coastal waters of Lianyungang, China (October 2020). Briefly, *N. scintillans* single cell was isolated with a large-diameter pipette based on the morphology and maintained in sterilized seawater (salinity of 30‰), which was prepared by filtration (0.22 μm filter) and autoclaving of *in situ* seawater. The culture was incubated at 20°C under light (100 μE m^–2^ s^–1^, 14: 10 h light–dark cycle) and with *Tetraselmis subcordiformis* as the prey (Wu et al., 1994; Luo et al., 2022).

To inhibit the growth of excess bacteria in the algae, *N. scintillans* cultures of 100 mL were pretreated with an antibiotic mixture of 100 μL ampicillin (200 mg/mL), kanamycin (100 mg/mL), and streptomycin (100 mg/mL) before the experiment. In preliminary experiments, this antibiotic mixture did not adversely affect the growth of *N. scintillans*.

### Isolation, screening, and identification of algicidal bacteria

We collected surface seawater from a phytoplankton bloom in the coastal waters of Lianyungang, China (34°46’57"N, 119°27’8"E) (Zhang et al., 2022). The water samples were serially diluted 10-fold with sterilized seawater, and 0.2 mL of each 10-fold serially diluted dilution was spread onto 2216E (10 g L^–1^ of tryptone, 2 g L^–1^ of yeast extract, 0.2 g L^–1^ of ferrous sulfate, and 1.0 L seawater) agar plates and incubated at 28°C for 48 h. The single colonies with significant differences in morphology were selected to obtain pure strains.

In order to screen the strains with algicidal activity against *N. scintillans*, we conducted pre-experiments in 24-well plates. All the purified strains were inoculated in 100 mL 2216E medium and cultured at 28°C, 180 rpm to their exponential growth period (OD_600_: 0.6–0.8). The bacterial culture was inoculated into 1 mL (10 cells mL^–1^) of algae cultures at 2 and 5% volume ratio, and an equal volume of sterilized 2216E medium and natural seawater was added to the algal cultures as control and blank groups, respectively. Bioassays were performed in triplicate, and all experiments were conducted during the light phase of the light cycle (20°C). The algal cells were counted at 4, 8, and 12 h to calculate the algicidal activity as follows:

Algicidalactivity(%)=(Nc-Nt)/Nc×100

where Nc and Nt represent the cell concentrations of *N. scintillans* measured in the control and experimental groups, respectively.

The cell density of *N. scintillans* was counted using a macroscopic observation because the cells are large enough (∼200–2,000 μm in diameter) to be seen by the naked eye (Zhang et al., 2020b). Gently shake the algal culture and collect 1 mL of culture for cell counting. Counting was performed three times in triplicate.

The morphological changes of algae cells were also examined and recorded under an inverted microscope to confirm the algicidal effect. The most effective and stable algicidal strain was selected for follow-up experiments.

Individual colonies of the screened algicidal bacterium were picked in 50 μL ddH_2_O (double distilled water), incubated at 100°C for 10 min, and then quickly transferred to ice. The bacterial lysate was taken as a template, and its 16S rDNA was amplified by PCR using the universal primers 16S-27F (5′-AGAGTTTGATCMTGGCTCAG-3′) and 16S-1492R (5′-TACGGYTACCTTGTTACGACTT-3′) for bacterial identification (DeLong, 1992). PCR amplification was performed in a 25 μL amplification volume, which contained 1 μL of each primer (10 μM), 2 μL of template, 12.5 μL of 2 × *Accurate Taq* Master Mix (Accurate Biology, China), and 8.5 μL ddH_2_O. An initial denaturation period of 95°C for 5 min, followed by 30 cycles of 94°C for 1 min, 55°C for 1 min, 72°C for 2 min, and the final extension at 72°C. Then the amplicons were sent to MAP (MAP Biotech, Shanghai, China) for sequencing. The obtained sequences were compared with those from the database for homology analysis using BLAST from the National Center for Biotechnology Information (Altschul et al., 1990). Multiple 16S rDNA sequences were aligned using ClustalW (Thompson et al., 2003). After removing the variable regions at both ends, 1,216 nucleotides positions were selected for phylogenetic analysis using the maximum-likelihood method with the Tamura-Nei model (1,000 bootstrap replications) in the MEGA 7.0 program (Kumar et al., 2016).

### Algicidal activity of LD-B6

Among 45 isolates, strain LD-B6 was chosen for further analysis. The LD-B6 strain was inoculated in 100 mL 2216E medium and cultured at 28°C, 180 rpm to their exponential growth period to obtain the LD-B6 cultures (Supplementary Figure 1). In order to assess the algicidal activity of different final concentrations of LD-B6 against *N*. *scintillans*, LD-B6 cultures were added to the pretreated (antibiotic-treated) algal cultures with different volume ratios (0.5, 1, 2, and 5%). To elaborate, 0.25, 0.5, 1, and 2.5 mL of bacterial cultures were added to 50 mL (10 cells mL^–1^) of the pretreated algal cultures. The same volume of sterilized seawater and 2216E medium was added to 50 mL of *N. scintillans* cultures as blank and control, respectively. All treatments were performed in triplicate and conducted during the light phase of the light cycle (20°C). The algal cells were counted after 4, 8, and 12 h of treatment, and the algicidal activity was calculated as described above.

To investigate the algicidal effect of LD-B6 on different densities of algal cultures, a 2% volume of cell-free supernatant of LD-B6 was added to 50 mL of the pretreated *N. scintillans* algal cultures with initial densities of 10, 30, and 50 cells mL^–1^, respectively. Also, a 2% volume of sterilized 2216E medium was used as a control. All experiments were performed in triplicate and conducted during the light phase of the light cycle (20°C). The algal cell concentration was counted at 12 h, and the algicidal activity was calculated as described above.

### Analysis of the algicidal mechanisms of LD-B6

To study the algicidal mode of LD-B6 against *N. scintillans*, the bacterial culture was treated in different ways: first, strain LD-B6 was inoculated in 100 mL of sterilized 2216E medium and cultured at 28°C, 180 rpm to their exponential growth phase to obtain the bacterial cultures, and then 10 mL bacterial cultures were collected and centrifuged at 12,000 rpm for 10 min. The collected supernatant was filtered with a 0.22-μm membrane to obtain the cell-free supernatant. The remaining cells were washed thrice with sterilized seawater and resuspended in 10 mL of sterilized seawater to obtain bacterial cells. The bacterial culture, cell-free supernatant, and bacterial cells were added to 50 mL (10 cells mL^–1^) of the pretreated algal cultures at the optimum concentration of 2% (*v/v*) to investigate the algicidal activity of these fractions. In addition, the same amounts of sterilized 2216E medium were added to the algal culture as the experimental control. All experiments were performed in triplicate and conducted during the light phase of the light cycle (20°C). The algal cells were counted after 4, 8, and 12 h of treatment, and the algicidal activity was calculated as described earlier.

### Stability of the LD-B6 cell-free supernatant

To examine the stability of the cell-free supernatant of strain LD-B6, the effect of different treatments on algicidal activity was investigated. The LD-B6 cell-free supernatant was incubated at different temperatures of −80, −20, 0, 15, 30, 60, and 100°C for 2 h, and then thawed or cooled to room temperature. The LD-B6 cell-free supernatant was adjusted to pH 3, 8, and 12 using NaOH or HCl and then adjusted back to the initial pH of 8 after 2 h. The supernatants treated in these two ways were inoculated into 50 mL (10 cells mL^–1^) of the pretreated algal cultures at 2% (*v/v*). All treatments were performed in triplicate and conducted during the light phase of the light cycle (20°C). The algicidal activity was calculated at 12 h to test the heat and acid-base tolerance of the cell-free supernatant. The algal cell count and the algicidal activity were computed as described above.

LD-B6 cell-free supernatant tubes were then put in liquid nitrogen for 5 min. They were taken it out and thawed in a thermostat water bath at 65°C, and this freeze-thaw step was repeated three times. The supernatant was cooled to room temperature and inoculated into 50 mL (10 cells mL^–1^) of the pretreated algal cultures at 2% (*v/v*). All experiments were performed in triplicate and conducted during the light phase of the light cycle (20°C). The algal cells were counted at 4, 8, and 12 h to calculate the algicidal activity. The algal cell counting and algicidal activity computations were as described above.

### Algicidal activity of LD-B6 on several HABs species

The algicidal efficiency of strain LD-B6 against typical HAB species was tested, including dinoflagellates (*N. scintillans* CCMA-LYG006, *Prorocentrum micans* CCMA-NT010, *Gymnodinium impudicum* CCMA-LYG009, and *Heterocapsa steinii* CCMA-LYG002), raphidophytes (*Heterosigma akashiwo* CCMA-LYG001), and diatoms (*Skeletonema costatum* CCMA277). Among them, *P. micans* was isolated from Nantong, China. *S. costatum* was provided by the Center for Collection of Marine Algae at Xiamen University. The rest strains were isolated from Lianyungang, China. Among them, *S. costatum* was cultured in the F/2 medium, and the other algae were cultured in the F/2-Si medium (Guillard, 1975). All algal cultures were incubated at 20°C under a 14: 10 h light–dark cycle with a photon flux of 100 μE m^–2^ s^–1^.

Flasks containing 50 mL of each species in an exponential growth period were inoculated with LD-B6 cultures at a final volume ratio of 2% (*v/v*). The same amounts of sterilized 2216E medium were used as controls. All treatments were performed in triplicate and incubated at 20°C. Algal cell concentrations were counted after 24 h. The cell concentration of *N. scintillans* was determined using macroscopic observations, and the algal cell density of other cultures was counted using a Sedgwick-Rafter chamber under a microscope.

### Algicidal powder production and application

Here, sawdust was selected as a carrier for LD-B6 immobilization. Briefly, sawdust was sterilized twice, dried, and used as the carrier. The carrier was added to the bacterial culture in the stable growth phase of LD-B6 at a mass fraction of 15% and then placed at 28°C and 180 rpm for co-incubation for 6 h to facilitate the sufficient adsorption of the sawdust. The un-adsorbed bacterial culture was removed by a screen mesh, and the adsorbed sawdust was placed on a glass petri dish and freeze-dried for 24 h. The lyophilized powder was stored at 4°C.

To assess the effectiveness of algicidal powder, 0.1 g of powder was added to 50 mL (10 cells mL^–1^) of the pretreated algal cultures, and the same volume of sterilized sawdust was added to 50 mL of *N. scintillans* cultures as control. All treatments were performed in triplicate and conducted during the light phase of the light cycle (20°C). The algal cells were counted at 12 h, and the algicidal activity was calculated as described above.

### Statistical analyses

All experiments were performed in triplicate, and data were presented as mean ± standard deviations. Statistical analyses were performed using SPSS16.0 software, and the significant differences among treatments in this study were analyzed by one-way ANOVA (*p* < 0.05).

## Results

### Screening and identification of algicidal bacteria

**FIGURE 1:**
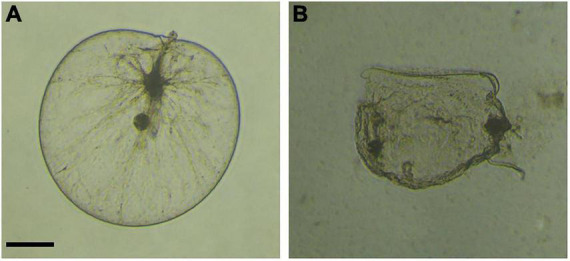
Microscope images of *Noctiluca scintillans* when co-incubated with strain LD-B6 at **(A)** 0 h, **(B)** 12 h. Scale bars = 100 μm.

After isolation and purification, a total of 45 strains were initially obtained. Among them, 11 strains showed algicidal activity against *N. scintillans*, and the LD-B6 strain had the strongest algicidal activity in the experiments with 2 and 5% volumes (Supplementary Figure 2). At the same time, microscopic imaging confirmed the effective algicidal activity of strain LD-B6. As shown in Figure 1 and Supplementary Video 1, the cell wall of *N. scintillans* began to break (6 h), and the contents flowed out after a period of co-culture of the bacterial culture and the algal culture, and then the rupture expanded continuously. Thus, the strain LD-B6 was selected for further study.

**FIGURE 2:**
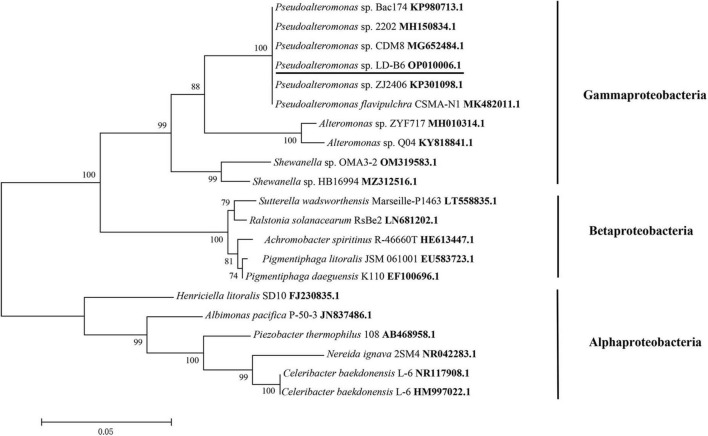
Phylogenetic tree of algicidal bacterial strain LD-B6 based on 16S rDNA sequence. Support of nodes > 50% is shown. Bar 0.05 means a nucleotide substitution rate of 0.05.

The 16S rDNA gene of LD-B6 was amplified and sequenced (GenBank accession number: OP010006.1). The 16S rDNA sequence of this strain showed the highest similarity (100%) to that of *Pseudoalteromonas* sp. CF1 (accession number KX570621). Meanwhile, in phylogenetic analysis, strain LD-B6 formed a monophyletic group with *Pseudoalteromonas* sp., corresponding to the Gammaproteobacteria subclass (Figure 2). This result indicated that strain LD-B6 belonged to *Pseudoalteromonas* sp., and it was designated as *Pseudoalteromonas* sp. LD-B6.

### Algicidal effects of LD-B6 on *Noctiluca scintillans*

**FIGURE 3:**
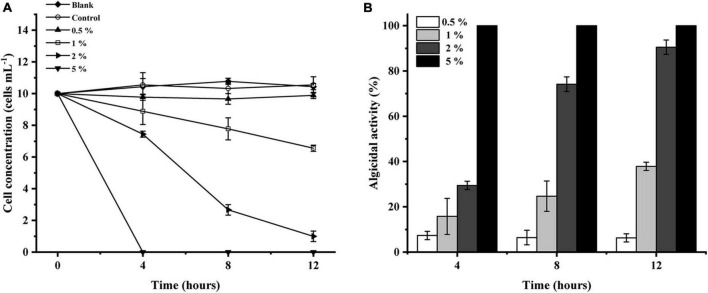
Algicidal activities of different concentrations of LD-B6 culture on *Noctiluca scintillans* based on **(A)** algal cell concentration and **(B)** algicidal activity. The error bars represent standard deviations (*n* = 3).

The algicidal activity of LD-B6 against *N. scintillans* at different concentrations was examined to determine the optimum final volume of the bacterial culture for further experiments. As shown in Figure 3B, LD-B6 at 1, 2, and 5% final volume activity have a certain algicidal effect against *N. scintillans*, and its algicidal activity increased in a concentration- and time-dependent manner, reaching 37.9 ± 1.8, 90.5 ± 3.2, and 100% at 12 h, respectively. However, the 0.5% treatment group only inhibited the growth of *N. scintillans* and did not kill algal cells (Figure 3A). Thus, the algicidal effect of strain LD-B6 against *N. scintillans* was concentration-dependent. As the algicidal activity in the 2% treatment group was higher than 90% at 12 h, this dose was chosen for further studies.

**FIGURE 4:**
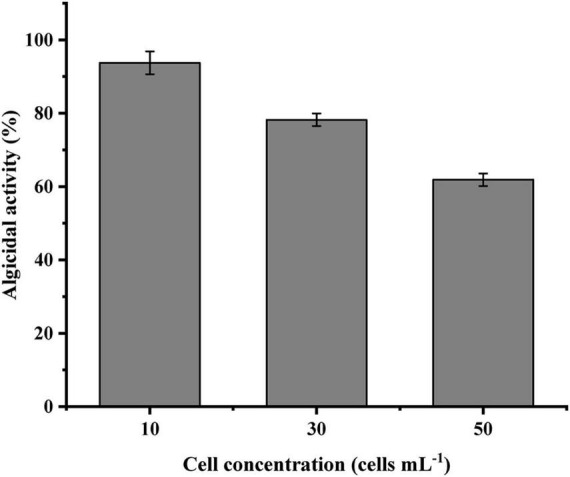
Algicidal effect of strain LD-B6 at different *Noctiluca scintillans* initial cell densities. The error bars represent standard deviations (*n* = 3).

The cell-free supernatant of LD-B6 was added to *N. scintillans* cultures at different initial densities to examine its algicidal activity. As shown in Figure 4, the algicidal effect of the cell-free supernatant was the highest when the initial density was 10 cells mL^–1^, followed by 30 and 50 cells mL^–1^at algicidal activity of 93.8 ± 3.1, 78.2 ± 1.7, and 61.9 ± 1.7%, respectively. Generally, the algicidal activity of LD-B6 against *N. scintillans* decreased with the initial density of the algal culture increasing.

### Algicidal mechanisms of LD-B6 on *Noctiluca scintillans*

**FIGURE 5:**
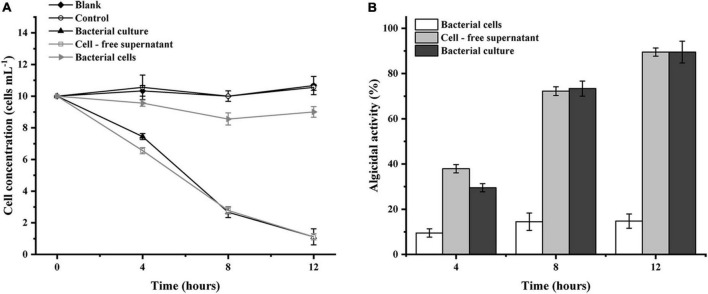
Algicidal activities of different fractions of LD-B6 culture on *Noctiluca scintillans* based on **(A)** algal cell concentration and **(B)** algicidal activity. The error bars represent standard deviations (*n* = 3).

To initially investigate the algicidal mechanisms of strain LD-B6, we added different fractions of bacterial cultures to *N. scintillans* cultures. The strain culture and cell-free supernatant showed an algicidal activity of 89.5 ± 1.8 and 89.5 ± 4.8%, respectively, after treatment for 12 h, while the algicidal effect of the bacterial cells was only 14.7 ± 3.2% and not obvious (Figure 5). The algicidal effect of the supernatant was significantly better than that of the bacterial cells themselves. It is presumed that strain LD-B6 lyses algal cells by secreting active compounds into the cell-free supernatant, and therefore strain LD-B6 lysed *N. scintillans* indirectly.

### Stability of the LD-B6 filtrate

**FIGURE 6:**
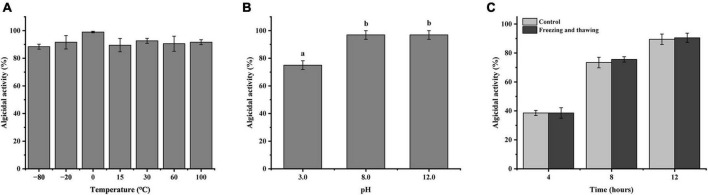
Algicidal effect of strain LD-B6 cell-free supernatant at different temperatures **(A)**, pH **(B)** conditions, and repeated freeze-thaw **(C)** treatment against *Noctiluca scintillans*. The error bars represent standard deviations (*n* = 3). Different letters a, b represent significant differences between groups (*p* < 0.05).

Under different temperature treatment conditions, the algicidal effect of strain LD-B6 compounds on *N. scintillans* is shown in Figure 6A. Although the algicidal activity was slightly different among treatments, there was no significant difference. It shows that the algicidal compounds of strain LD-B6 have definite thermal stability. Incubation at different pH values for 2 h had an effect on the algicidal activity of the cell-free supernatant of strain LD-B6. The algicidal compounds of strain LD-B6 were sensitive to acid, and their algicidal activities were significantly decreased under acidic conditions. In contrast, the algicidal effect of the cell-free supernatant was stable under alkaline conditions (Figure 6B). Repeated freezing and thawing had almost no effect on the algicidal activity of the LD-B6 cell-free supernatant, and there was no significant difference in the algicidal effect between the control and the experiments (Figure 6C).

### Algicidal activity of LD-B6 on several HABs species

**FIGURE 7:**
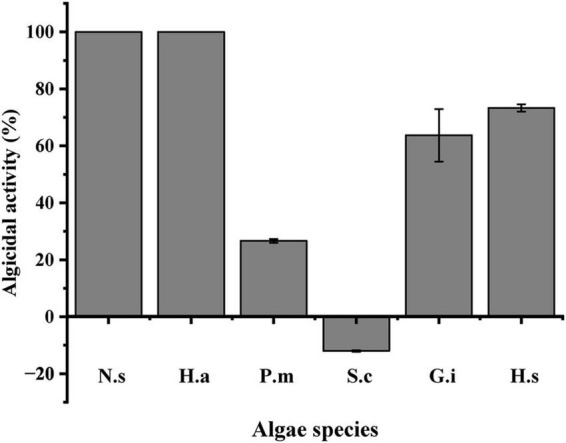
Algicidal activity of bacterial strain LD-B6 on representative HABs species. N.s, *Noctiluca scintillans*; H.a, *Heterosigma akashiwo*; P.m, *Prorocentrum micans*; S.c, *Skeletonema costatum*; G.i, *Gymnodinium impudicum*; H.s, *Heterocapsa steinii*. The error bars represent standard deviations (*n* = 3).

The algicidal activity of LD-B6 culture against six different HABs species is shown in Figure 7. Within 24 h of incubation, the LD-B6 culture showed the strongest algicidal effect on *N. scintillans* and *H. akashiwo*, and the algicidal activity reached 100%. It also had a strong algicidal activity on *H. steinii* and *G. impudicum*, with the algicidal activity of 73.3 ± 1.3 and 63.7 ± 9.2%, respectively. A weak effect on *P. micans* (26.7 ± 0.68%) was observed. In contrast, the growth of *S. costatum* was slightly promoted by strain LD-B6.

### Algicidal powder production and application

Algicidal powder product status is shown in Supplementary Figure 3. The algicidal powder of LD-B6 was added to *N. scintillans* cultures to assess its algicidal effect. As shown in Supplementary Figure 4, the algicidal activity of 100% at 12 h with 0.2% final volume of algicidal powder. This result indicated that the developed algicidal powder has a strong algicidal effect and that the production method is feasible.

## Discussion

During algal bloom formation and decline, the populations and dominance of marine bacteria change significantly, especially in the period of extinction (Yang et al., 2015). More importantly, some marine bacteria play an important role at the end of the HABs (Li et al., 2018). Therefore, the research on the relationship between bacteria and algae in controlling algal blooms has become a hot topic. However, the interactions between bacteria and the alga *N. scintillans* have received limited attention. In this work, a bacterial strain LD-B6 with algicidal activity against *N. scintillans* was isolated. This strain was identified by 16S rDNA sequencing to belong to *Pseudoalteromona*s in the class Gammaproteobacteria (Figure 2). Several investigations have documented that Gammaproteobacteria is the dominant bacteria during HABs, and the dominant population of marine bacteria exhibit a succession from Alphaproteobacteria to Gammaproteobacteria during the late-blooming phase, indicating that they may influence algal bloom dynamics (Teeling et al., 2012; Zhang et al., 2016; Li et al., 2018). *Pseudoalteromonas* sp. is one of the most frequently reported and abundant algicidal bacterial species in the ocean. It has been documented that bacteria of this genus show algicidal effects against phytoplankton, including Dinophyceae, Raphidophyceae, and Bacillariophyceae (Lovejoy et al., 1998; Lee et al., 2000; Zhang et al., 2022). Therefore, this isolate may be involved in the regulation of algal blooms.

Strain LD-B6 had a strong algicidal effect on *N. scintillans*. The normal cells of *N. scintillans* are nearly spherical, with a full and smooth surface. Twelve hours after LD-B6 bacterial cultures were added, the cells showed obvious folds and atrophy under the microscope (Figure 1). In addition, the algicidal activity of LD-B6 on *N. scintillans* was also recorded by an inverted microscope (Supplementary Video 1), which indicates that the cell wall of *N. scintillans* was the main target of LD-B6. At present, a number of algicidal bacteria have been proven to lyse algal cells by degrading cell wall polysaccharides (Kim et al., 2009; Wang et al., 2010; Li et al., 2022). Hydrolytic enzymes such as amylase, cellulase, and xylanase are the causes of such polysaccharide degradation (Li et al., 2022). These results enrich the understanding of the mechanism of algal cell lysis by LD-B6.

It has been reported that the algicidal activity of bacteria depends on bacterial concentration and algal cell density (Tian et al., 2012; Kong et al., 2020; Al-Hakimi et al., 2020). For instance, Shao et al. (2015) found that *Bacillus* sp. B50 showed an algicidal activity when the bacterial concentration reached 1.9 × 10^6^ CFU/mL, while the algicidal activity was lost at a cell concentration below 1.9 × 10^5^ CFU/mL. Yang et al. (2020) indicated that the higher the initial concentration of algal cells, the poorer the algicidal activity of the *Ponticoccus* sp. CBA02. Similar to these previous reports, the algicidal effect of LD-B6 against *N. scintillans* also reflects these patterns. When a low concentration of bacterial culture was inoculated into the algal culture, it only inhibited the growth of *N. scintillans*, while the high concentration of bacteria cultures showed a strong algicidal effect (Figure 3). In addition, we also found that the algicidal effect of LD-B6 decreased with the increase in the initial density of algal cultures (Figure 4). These observations can be explained as that with the increase in the bacterial concentration, there are more algicidal compounds per unit volume, and the contact probability between algae cells and algicidal compounds is further increased, thus enhancing the algicidal effect. On the contrary, a high concentration of algal cells may reduce the concentration of algicidal compounds exposed to individual algal cells, thereby affecting the algicidal activity. Therefore, the control of HABs can entail the inoculation of a certain concentration of algicidal bacteria at the initial stage of bloom, or the concentration of algicidal bacteria can be adjusted according to the development stage of an algal bloom.

Algicidal bacteria generally exert two main strategies for lysing algae: direct and indirect modes (Azam, 1998). In this study, the cell-free supernatant of strain LD-B6 showed a strong algicidal effect against *N. scintillans*, indicating an indirect manner of targeting. This is on the same lines as algicidal bacteria belonging to *Pseudoalteromonas* spp. previously reported (Wang et al., 2020), which lyse target algae by releasing extracellular substances. The characteristics of algicidal compounds are influenced by various factors such as temperature and pH (Fu et al., 2011; Zhang et al., 2022). Bai et al. (2011) reported that the compounds of *Brevibacterium* sp. BS01 were heat tolerant and stable in acidic or alkali conditions, indicating these compounds are not proteins. In addition, the algicidal compounds of *Pseudoalteromonas* sp. SP48 are tolerant to heat but unstable under acidic conditions (Su et al., 2007). Our findings showed that the algicidal compounds of LD-B6 had freeze-thaw stability, were heat tolerant, and were stable under alkaline conditions, while acidic conditions had a significant effect on the algicidal activity (Figure 6). As described in reviews, algicidal bacteria can produce several kinds of algicidal substances, including ectoenzymes (e.g., serine protease and extracellular agarase), pigments (e.g., isatin and prodigiosin) (Meyer et al., 2017; Wang et al., 2020; Coyne et al., 2022). Based on the thermal stability of algicidal compounds secreted by strain LD-B6, it is unlikely that this compound is a protein. Alternatively, it is possible that algicidal compounds of LD-B6 are a pigment. Examples include an algicidal substance of *Pseudomonas* sp. C55a-2 was identified as a pigment (Sakata et al., 2011). Further, the properties, types, and extraction of algicidal compounds still need to be studied.

Different strains of algicidal bacteria likely show different degrees of specificity. Some bacteria are species-specific, lysing only specific algae. Shi et al. (2020) isolated a strain FDHY-CJ8, which had algicidal activity only on *S. costatum*, but almost no algicidal effect on other algae. Pokrzywinski et al. (2012) found that the algicidal bacterium *Shewanella* sp. IRI-160 showed selectivity toward dinoflagellates. Some strains have broad specificity and are able to lyse a wide range of algal species. For example, the bacterium YX04 has an algicidal effect on the taxa of Bacillariophyceae, Pyrrophyta, and Chrysophyta (Zhu et al., 2022). Highly species-specific or broadly specific algicidal bacteria are related to their type and secreted compounds (Meyer et al., 2017). Most bacteria in the free-living form are highly species-specific, and the bacteria in the particle-associated form are mostly broadly specific (Park et al., 2010). Strain LD-B6 showed varying degrees of algicidal effects against five of the six typical harmful algal species tested, including dinoflagellates and a raphidophyte (Figure 7), indicating its broad-spectrum algicidal ability. This potential of LD-B6 to control HABs, combined with the stability of the algicidal compounds as previously described, makes it potentially useful as a promising agent against HABs.

In recent years, more and more algicidal agents have been developed to control algal blooms. The use of carrier immobilization to improve the colonization activity of algicidal agents is an effective way to ensure the sustainability of the algicidal effect; sawdust is regarded as an ideal carrier because of its outstanding adsorption ability (Ye et al., 2022). In this study, strain LD-B6 was also immobilized, and sterile sawdust was used as a carrier to prepare an algicidal powder (Supplementary Figure 3). The results showed that the algicidal activity of the prepared powder was 100% (12 h), which was 9.5% higher than that of the bacterial culture at a final concentration of 2%. It may be that the immobilization by the carrier effectively improves the biomass of microbial agents, their resistance to water flow, and their tolerance to toxic compounds (Chen et al., 2003).

## Conclusion

The algicidal bacterium LD-B6, with strong algicidal activity against *N. scintillans*, was isolated from the coastal waters of Lianyungang, China. This bacterium lysed algae indirectly by secreting extracellular compounds, and these algicidal compounds are stable, indicating that they are not proteins. In addition, strain LD-B6 was broadly specific, and the preliminary development of an algicidal powder is also presented. These results documented the ability of strain LD-B6 and its algicidal compounds to control *N. scintillans* blooms and provide a foundation for their practical applications. In the future, it would be necessary to extract and analyze algicidal compounds and control them in a targeted manner according to the characteristics of the applied environment, such as solving the adaptation problems of salinity, temperature, pH, etc., to develop more efficient algicidal agents to control HABs.

## Data availability statement

The datasets presented in this study can be found in online repositories. The names of the repository/repositories and accession number (s) can be found in the article/Supplementary material.

## Author contributions

JW and NJ conceived the study, performed the experiment, and prepared the manuscript. XY, MX, and YCh completed the experiment. HG, YCa, and XS prepared the manuscript. All authors have read and agreed to the published version of the manuscript.

## Supplementary material

Supplementary Figure 1 Growth curve of strain LD-B6.

Supplementary Figure 2 The algicidal effects of different strains and concentrations on *Noctiluca scintillans*.

Supplementary Figure 3 Algicidal powder product status.

Supplementary Figure 4 Algicidal activity of algicidal powder of LD-B6 on *Noctiluca scintillans.*

Supplementary Video 1 Algicidal activity of LD-B6 on *Noctiluca scintillans*.
