# Supplementary material for: Isolation and characterization of a high-efficiency algicidal bacterium Pseudoalteromonas sp. LD-B6 against the harmful dinoflagellate Noctiluca scintillans
Source: Front Microbiol. 2022 Dec 22;13:1091561. doi: 10.3389/fmicb.2022.1091561 (PMC9814975; doi:10.3389/fmicb.2022.1091561)
Supplement: Supplementary Figure 1 — Growth curve of strain LD-B6. [file Data_Sheet_1.docx]

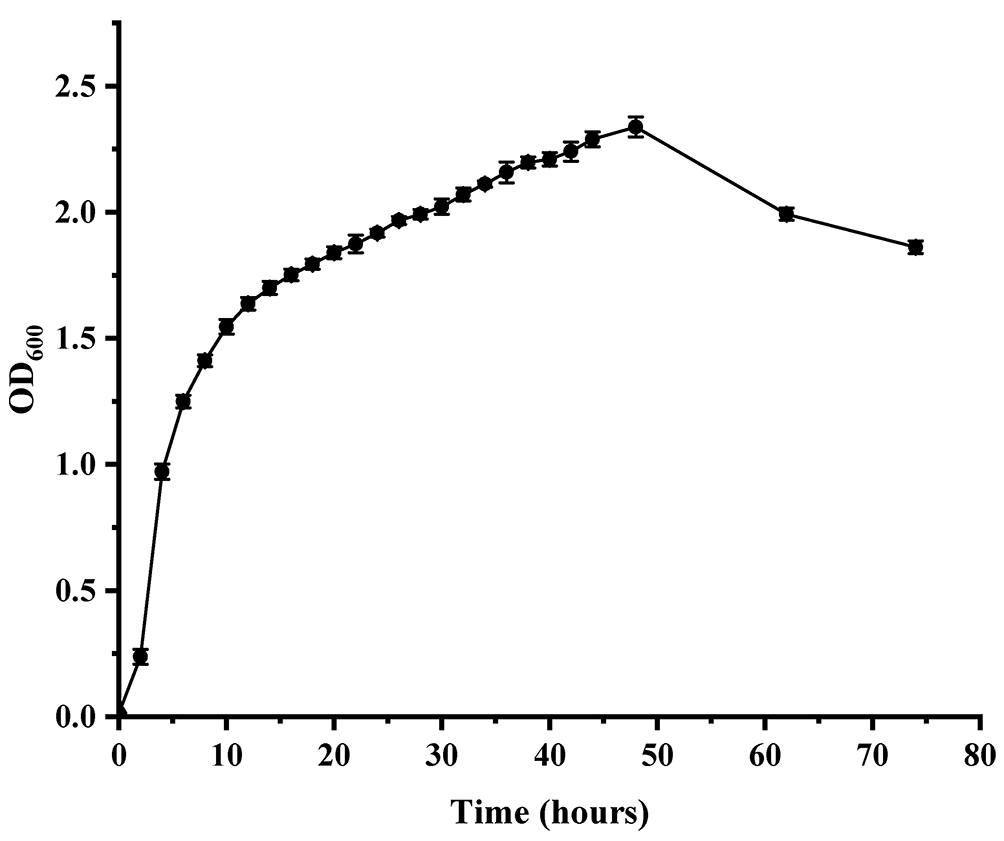


**Figure S1.** Growth curve of strain LD-B6.


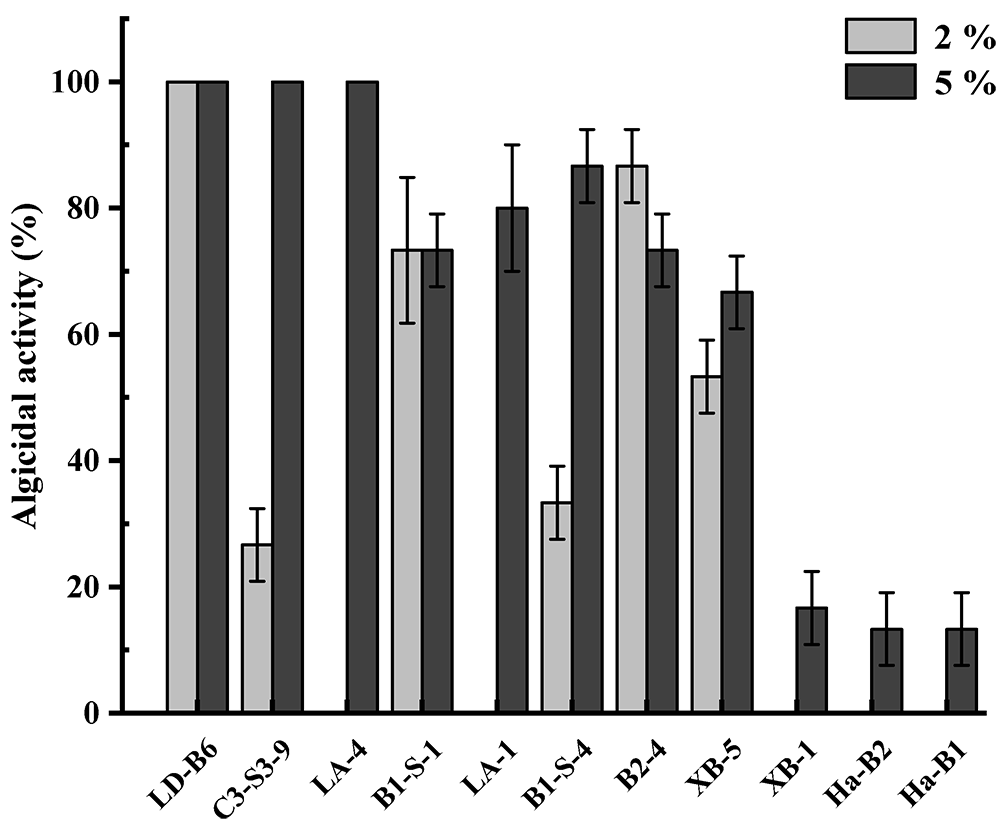


**Figure S2.** The algicidal effect of different strains and concentrations on *Noctiluca scintillans.*


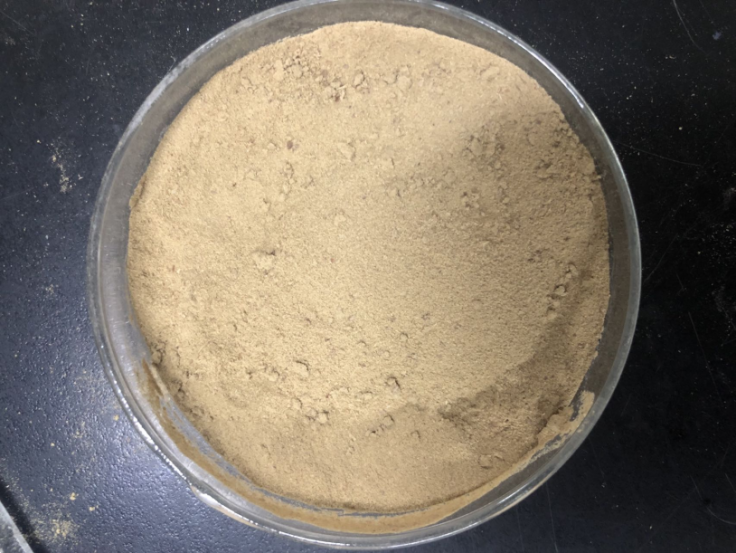


**Figure S3.** Algicidal powder product status.


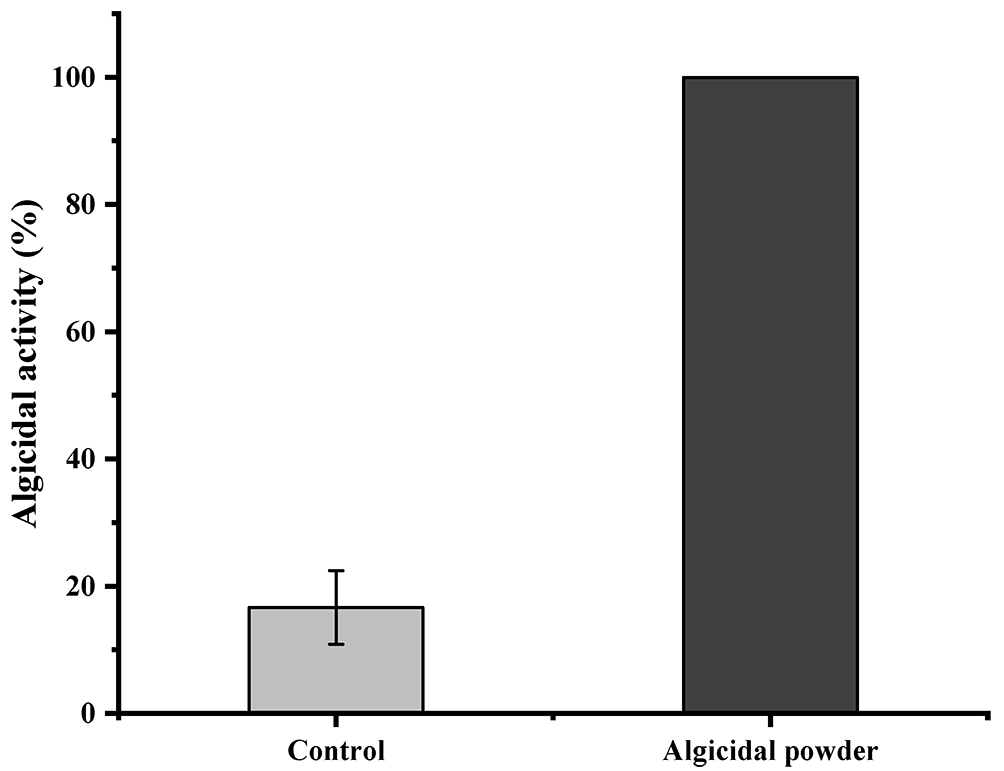


**Figure S4.** Algicidal activity of algicidal powder of LD-B6 on *Noctiluca scintillans.*
